# ROS-induced oxidative damage in lymphocytes ex vivo/in vitro from healthy individuals and MGUS patients: protection by myricetin bulk and nanoforms

**DOI:** 10.1007/s00204-020-02688-4

**Published:** 2020-02-27

**Authors:** Shabana Akhtar, Mojgan Najafzadeh, Mohammad Isreb, Lisa Newton, Rajendran C. Gopalan, Diana Anderson

**Affiliations:** 1 School of Chemistry and Biosciences, University of Bradford, Bradford, UK; 2 School of Pharmacy and Medical Sciences, University of Bradford, Bradford, UK; 3 Bradford Royal Infirmary (BRI), Bradford, UK

**Keywords:** Myricetin bulk forms and nanoforms, TBHP, hydrogen peroxide, Lymphocytes, MGUS patients, γ-H2AX

## Abstract

We investigated the protective role of myricetin bulk and nanoforms, against reactive oxygen species (ROS)-induced oxidative stress caused by hydrogen peroxide and tertiary-butyl hydro peroxide in lymphocytes in vitro from healthy individuals and those from pre-cancerous patients suffering with monoclonal gammopathy of undetermined significance (MGUS). The change in intracellular reactive oxygen species was measured once cells were treated with myricetin bulk forms and nanoforms with and without either hydrogen peroxide or tertiary-butyl hydro peroxide co-supplementation. The direct and indirect antioxidant activity of myricetin was spectrofluometrically measured using the fluorescent dye 2′,7′-dichlorofluorescin diacetate and using the Comet assay, respectively. Hydrogen peroxide (50 µM) and tertiary-butyl hydro peroxide (300 µM) induced a higher level of reactive oxygen species-related DNA damage and strand breaks. Addition of myricetin nanoform (20 µM) and bulk (10 µM) form could, however, significantly prevent hydrogen peroxide- and tertiary-butyl hydro peroxide-induced oxidative imbalances and the nanoform was more effective. Glutathione levels were also quantified using a non-fluorescent dye. Results suggest that myricetin treatment had no significant effect on the cellular antioxidant enzyme, glutathione. The current study also investigates the effect of myricetin on the induction of double-strand breaks by staining the gamma-H2AX foci immunocytochemically. It was observed that myricetin does not induce double-strand breaks at basal levels rather demonstrated a protective effect.

## Introduction

Oxidative stress is a major factor contributing towards the development of various illnesses and it is a key inducer of cellular lesions. It is triggered by a disparity between the production of reactive oxygen species (ROS) and the capacity of biological system to cleanse the reactive intermediates. ROS have been shown to induce proliferation in tumour cells and mediate the proliferation initiated by epidermal growth factor (EGF) or platelet-derived growth factor (PDGF) (Burdick et al. 2003; Chao-Wei Chen et al. 2004). Thus, antioxidants show an effective preventative effect against cancer development by reducing the ROS levels and inhibiting their production. In addition, intracellular ROS production has been associated with the mediation of the effects caused by anti-cancer drugs like taxol (Perkins et al. 2000; Varbiro et al. 2001). Hence data suggests a two-sided role of ROS. It has been suggested that cellular redox potential may be regulated through increasing glutathione (GSH) pools to the mitochondria, hence, preventing ROS formation and cellular injury caused by oxidative stress (Slikker et al. 2001).

Oxidative stress produced by tertiary-butyl hydro peroxide (TBHP) and hydrogen peroxide (H_2_O_2_) is well documented. TBHP is a toxic compound and causes extreme discomfort to various organs and systems (Zavodnik et al. 1988; Sarkar and Sil 2010; Bhattacharya et al. 2011). Exposure of cellular components to TBHP results in an increase of membrane permeability along with hyperpolarization. Reaction of TBHP with haemoglobin forms t-butoxyl radicals, which then initiate peroxidation by interacting with membrane lipids (Deuticke et al. 1987). Extensive lipid peroxidation leads to membrane disturbance (Benatti et al. 1982). Cellular antioxidant enzymes, like glutathione and l-ascorbate, to some level inhibit membrane disruption by scavenging the t-butoxyl radicals.

Myricetin (3,5,7,3′,4′,5′-hexahydroxyflavone), an important flavonol, is widely found in berries, fruit, and vegetables (Wang et al. 2010). A large body of data has been published regarding the antioxidant potential of myricetin, leaving no doubt that the compound is a strong antioxidant. It depicts antioxidant activity by scavenging ROS through oxidation of the three hydroxyl groups (3′,4′,5′-position) attached in its B ring (Chobot and Hadacek 2011). Substantial amounts of evidence suggest that myricetin shows other promising health beneficiary effects including chemo preventative, anti-diabetic, anti-cancer, anti-inflammatory, anti-allergic, and activity against cardiovascular diseases (Semwal et al. 2016). Studies have demonstrated that the physiological concentrations range of myricetin (5–10 µM) (Peng and Kuo 2003) could significantly inhibit the production of peroxynitrite-induced double-strand breaks (DSBs) (Chen et al. 2011) and protect against the oxidative damage caused in neurodegenerative disorders (Laabich et al. 2007; Shimmyo et al. 2008). Myricetin has been demonstrated to prevent the TBHP-induced chemiluminescence of mouse liver homogenates (Fraga et al. 1987) and oxidative stress in erythrocytes from Type-2 diabetic patients in vitro (Pandey et al. 2009). H_2_O_2_-induced oxidative stress and DNA strand breaks caused in human lymphocytes and human colonocyte cells have been demonstrated and shown to be reduced upon myricetin treatment (Dutie et al. 1997).

Although myricetin is a well-established antioxidant, little is known about its beneficial role in countering the TBHP-induced cytotoxicity in primary lymphocytes ex vivo in vitro of healthy individuals and monoclonal gammopathy of undermined significance (MGUS) patients. MGUS is an asymptomatic precursor (pre-cancerous) condition of multiple myeloma (MM) and it is well documented that almost every case of MM is headed by MGUS (Dhodapkar 2016). The aim of the present study was, therefore, to investigate the in vitro protective role of myricetin nanoparticles (MYR N) and myricetin bulk (MYR B) against TBHP-induced oxidative damage in lymphocytes from healthy individuals and MGUS patients. The effect of myricetin nanoforms and bulk forms against H_2_O_2_-induced damage, change in intracellular ROS due to oxidative stress caused by TBHP and change in GSH levels was investigated. The study also assessed the effect of myricetin nanoforms and bulk forms on DSBs at basal levels by analysing the gamma-H2AX protein expression immunocytochemically.

## Methodology

### Blood sample collection and ethics

The current project involving the use of human peripheral lymphocytes has been granted ethical approval by Leeds East Ethics Committee (IRAS Reference No.:12/YH/0464) and the University of Bradford’s Sub-Committee for Ethics in Research involving healthy Human Subjects (Reference No.: 0405/8). All peripheral blood samples were collected after informed consent from patients and healthy individuals. The research support and governance office of Bradford Teaching Hospitals NHS Foundation also agreed the research (REDA number 1202).

### Informed consent

The blood samples from healthy individuals and MGUS patients used in the current study were collected after obtaining informed consent from the participants and are listed in Tables 1 and 2, respectively. Exclusion criteria for human subjects enrolled in the study were anaemia, blood diseases or major disorders.

**Table 1:** Healthy blood samples

No	Age	Ethnicity	Gender	Smoking history	Family history
1	48	Caucasian	M	No	None
2	28	Caucasian	M	No	None
3	27	African	M	Yes	None
4	38	Caucasian	M	No	None
5	60	Caucasian	F	No	None
6	40	Arab	M	No	None
7	45	Caucasian	M	Yes	None
8	55	Caucasian	M	No	None
9	35	Caucasian	M	Yes	None
10	25	Caucasian	M	No	None
11	44	Asian	M	Yes	None
12	28	Caucasian	M	No	None
13	23	Caucasian	F	No	none
14	27	Caucasian	M	No	None
15	33	Arab	M	Yes	None
16	47	Asian	M	Yes	None
17	28	Caucasian	M	No	None
18	42	Asian	M	No	None
19	48	Asian	M	No	None
20	60	Asian	M	Yes	None

**Table 2:** Brief information about patient samples

No	Age	Ethnicity	Gender	Smoking history	Family history	Medical condition
1	79	Caucasian	M	No	None	MGUS
2	80	Caucasian	F	No	None	MGUS
3	78	Caucasian	M	No	None	MGUS
4	56	Caucasian	F	Yes	Leukaemia and brain Tumour	MGUS
5	77	Caucasian	M	No	None	MGUS
6	75	Caucasian	M	No	None	MGUS
7	80	Caucasian	M	No	Cancer Positive	MGUS
8	81	Caucasian	F	No	Bowel and stomach	MGUS
9	63	Caucasian	M	Yes	None	MGUS
10	55	Caucasian	M	No	None	MGUS
11	83	Caucasian	M	No	None	MGUS
12	63	Caucasian	M	Yes	None	MGUS
13	74	Caucasian	M	No	None	MGUS
14	63	caucasian	F	Yes	Arthritis	MGUS
15	66	Caucasian	F	No	Breast cancer	MGUS
16	52	caucasian	M	Yes	None	MGUS
17	83	Caucasian	M	No	None	MGUS
18	60	Asian	F	No	None	MGUS
19	75	Caucasian	F	No	Mastectomy	MGUS
20	69	Caucasian	M	No	Stomach and lung	MGUS

*M* male, *F* female, *MGUS* monoclonal gammopathy of unknown significance

### Preparation of myricetin bulk forms and nanoforms, concentration, solubility, and zeta potential of nanoparticles

Myricetin powder (˃ 96% purity) was purchased from Fisher Scientific, UK. Suspensions of myricetin bulk forms and nanoforms were made in an excipient mixture (containing 7% (w/w) solid loads of myricetin in a medium comprising of hydroxypropyl methylcellulose (HPMC) (0.5% w/w), sodium lauryl sulphate (SLS) (0.1% w/w), ethanol (0.8% w/w), polyvinylpyrrolidone (PVP) K-30 (0.5% w/w) and purified water). The suspensions were transferred to an amber glass bottles and stored at 4 °C for the research duration. The mean particle sizes and zeta potential (ZP) of myricetin bulk and nano in the stock solutions were measured using a Zetasizer Nano ZS-90 Model ZEN3600 (Malvern Instruments Ltd, UK) by photon correlation spectroscopy. The initial particle sizes of bulk and nano were 1737 and 161 nm, respectively and their ZP measurement was less than 1% indicating their good stability. The suspensions were also sonicated for 10 min before each use to avoid sedimentation and control aggregation.

### In vitro experimental design, preliminary treatment, and concentration range study in lymphocytes

**Fig. 1:**
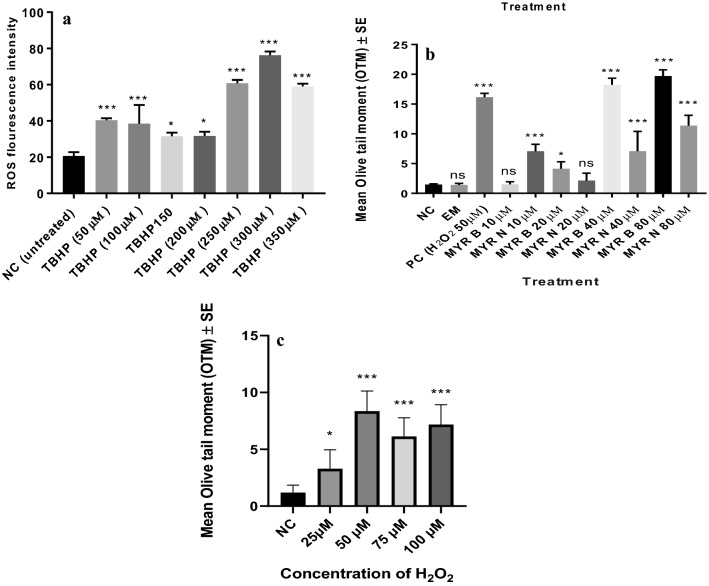
**a** Average of three independent experiments showing, change in intracellular ROS after treatment with different concentrations of TBHP in healthy lymphocytes. All groups were compared against the untreated group. ***P < 0.001, *P = 0.02. Results are expressed as mean ± SE. **b** Concentration dependent responses of MYR B and MYR N in healthy lymphocytes showing mean OTM. All data have been expressed as mean ± standard errors (SE) of experiments in healthy individuals, measuring 100 cells each. In vitro treatment of healthy lymphocytes with different concentrations of MYR B and MYR N in the Comet assay has shown that MYR B (10 µM) and MYR N (20 µM) produced least levels of the DNA damage which was comparative with the negative control (NC). Therefore, these optimum concentrations have been used throughout the study. Other treatment groups included, excipient mixture (EM) (0.1%) and PC (H_2_O_2_ 50 µM). The stars indicate significant difference between NC and various treatment groups (*P* < 0.05 significantly different), *ns* indicates not significant, ***P < 0.001, *P < 0.01. **c** Concentration-dependent responses of H_2_O_2_ in healthy lymphocytes showing mean OTM. All data have been expressed as mean ± standard errors (SE) of experiments in healthy individuals, measuring 100 cells each. In vitro treatment of healthy lymphocytes with different concentrations of H_2_O_2_ in the Comet assay has shown that 50 µM of H_2_O_2_ produced most significant levels of the DNA damage compared to the NC. Therefore, this optimum concentration has been used throughout the study as a PC (*P* < 0.05 was considered significantly different), *ns* indicates not significant, ***P < 0.001, *P < 0.01

TBHP is a known intracellular stress inducer hence; increase in ROS production was used as a factor to choose the optimum concentration of TBHP for 0–60 min. The concentration dependent responses of TBHP are shown in Fig. 1a. TBHP has induced significant levels of ROS at each determined concentration after 60 min of treatment. However, at 300 µM, its activity was at highest when compared to the untreated group.

Flavonoid compounds have this ability to cause adverse effects at slightly different concentrations; therefore, we used the safer concentrations with no significant genotoxicity. Based on the dose–response studies, 10 µM for MYR B and 20 µM for MYR N were optimal doses with no cellular toxicity (Fig. 1b).

The optimal concentration of H_2_O_2_ (50 µM) used throughout the study as the positive control and for co-supplementation in the Comet assay was determined by the dose response (1c). Untreated lymphocytes were set as the negative control (NC). The optimal concentrations for TBHP and H_2_O_2_ were used as positive controls (PC). After determining the optimum doses for the test chemicals, PCs were simultaneously added to lymphocytes in suspension with either MYR B or MYR N in co-supplementation studies.

### Confounding factors

The potential effects of the confounder such as smoking, age, ethnicity, and gender on results were analysed using the Comet assay. However, myricetin had no significantly different effect on any of these factors (Table 3).

**Table 3:** The effect of confounding factors in healthy individuals and MGUS patients using OTM

Subject	Treatment group	Smoker	Non-smoker	Asian	Caucasian	Male	Female	Young	Old
		OTM	OTM	OTM	OTM	OTM	OTM	OTM	OTM
Healthy individuals	NC	1.1	0.7	0.7	0.7	1.2	0.8	2.7	4.1
	PC (H_2_O_2_ 50 µM)	8.9	10.7	11.8	8.9	8.1	13.5	6.7	10.3
	MYR B (10 µM)	2.0	1.49	1.1	2.0	1.5	1.3	3.1	6.0
	MYR N (20 µM)	1.5	0.8	1.4	0.89	2.0	1.2	1.6	3.05
MGUS patients	NC	3.6	2.8	4.0	2.5	2.2	2.6	3.5	3.2
	PC (H_2_O_2_ 50 µM)	9.0	8.7	15.5	6.2	6.4	5.4	9.5	11.9
	MYR B (10 µM)	2.5	3.4	6.9	3.8	2.6	3.3	3.9462	4.5
	MYR N (20 µM)	2.3	1.7	4	2.9	1.0	1.8	2.1384	3.1

### MTT assay

The cytotoxicity of chemicals was determined by 3-(4,5-dimethylthiazol-2-yl)-2,5-diphenyltetrazolium bromide (MTT) dye absorbance. Isolated lymphocytes (10^4^) were maintained in complete medium overnight in 96-well plates followed by treatment the next day. After the exposure time (1 and 24 h) was over, 10 µl of MTT dye (Fisher Scientific, UK) (5 mg/ml) was added to each well and incubated for another 4 h at 37 °C in the dark. After this, medium was aspirated and 200 µl of DMSO (Fisher Scientific, UK) was added to each treatment and absorbance was read at 570 nm.

### The Comet assay for determination of DNA damage

Lymphocytes were treated with MYR B (10 µM) and MYR N (20 µM) in combination with H_2_O_2_ (50 µM) for 1 h. The cell suspension was centrifuged at 3000 rpm (1000 g). The supernatant was removed and the pelleted cells were subjected for the Comet assay as previously defined with some changes (Singh et al. 1988; Tice et al. 2000; OECD 2016; Anderson et al. 2014; Azqueta and Dusinska 2015).

### Measurement of ROS accumulation in isolated lymphocytes

Isolated lymphocytes were grown in a 96-well plate overnight. Cells were washed with phosphate buffer saline (PBS) prior to addition of various chemical treatments in PBS and incubated for 1 h at 37 °C in the presence of 5% CO_2._ Once incubation was over, the cells were washed again with PBS following the loading of a fluorescent probe, DCFDA dye (Abcam, UK) into each well and incubated again for 45 min under the same conditions. Then dye was replaced by 100 µl of PBS and fluorescence was measured at 485/535 nm using Promega Glomax explorer version 2.4. Data were analysed using the Graph Pad Prism 7.02.

### Assay of cellular enzyme and total thiol content

Reduced glutathione (GSH) levels and oxidised glutathione (GSSG) contents in experimental and normal lymphocytes were analysed using the GSH/GSSG Ratio Detection Assay Kit (Fluorometric—Green) (Abcam, UK) according to the manufacturer protocol. Briefly, isolated lymphocytes were harvested overnight in 6-well plate then treated with chemicals for 1 h. Cells were washed with cold PBS and re-suspended in 100 µl of cold lysis buffer supplemented with 10 µl of protease inhibitors then thoroughly mixed by pipetting and centrifuged at 400*g* for 5 min to remove insoluble. The supernatant (sample) was collected and kept on ice for further use. 50 µl of each GSH and GSSG standards (kit components) were added to 96-well plates in duplicates. For GSH detection, 50 µl of the GSH assay mixture (GAM) (provided with the kit) was added to each GSH standard and sample. For GSSG detection, total GSH assay mixture (TGAM) was added to each GSSG standard and sample. Incubated for 60 min at room temperature in the dark and then fluorescence was measured at 490/520 nm using Promega Glumax explorer version 2.4. Data were analysed using Graph Pad Prism 7.02.

### Determination of histone-2AX (H2AX) phosphorylation (γ-H2AX) levels using Immunocytochemistry

The method was based on that of Schmid et al. (2010) and previously followed in our lab (Laubenthal et al. 2012). A total of 100 cells per treatment were examined under the fluorescence microscope connected with a CCD camera (Nikon Digital Sight DS-SMC, Surrey, UK) for gamma-H2AX foci expression, a marker of DNA double-strand breaks. Untreated cells were used as the negative control, whereas doxorubicin (50 µM) was used as the positive control. Each experiment was done in triplicate with both controls included.

### Statistical analysis

Results were presented as mean ± SE and differences between the groups were analysed by one-way and two-way analysis of variance (ANOVA) using Graph Pad prism 7 software. Values of *p* < 0.05 were considered significantly different.

## Results

### Cytotoxicity of chemicals in lymphocytes

**Fig. 2:**
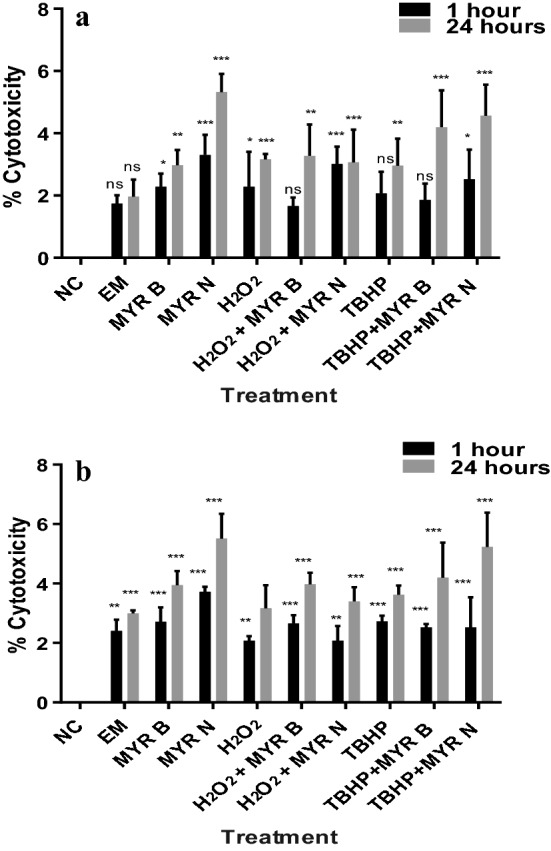
% Cytotoxicity in 2 × 10^4^ cells of various test treatments in lymphocytes from healthy volunteers (**a**) and MGUS patients (**b**) using MTT assay. Overall values were less than 5% which is not considered as cytotoxic. Error bars show mean values ± SE, *n* = 3. *P = 0.03, **P = 0.002, ***P = 0.0002, *ns* not significant

Cytotoxicity of test chemicals was determined by culturing lymphocytes from healthy individuals and MGUS patients and measuring the mean absorbance at 1 and 24 h using the MTT dye. Results from the MTT assay showed that the test chemicals used in this study exhibited non-significant level of cytotoxicity (which was approximately 5% lower compared to the untreated cells) occurred only after 24 h treatment, for all the treatment groups assessed in lymphocytes from both healthy and patient group (Fig. 2a, b).

### Effect of MYR N & B on hydrogen peroxide (H_2_O_2_)-induced DNA damage in lymphocytes from healthy vs patient group using the Comet assay

**Fig. 3:**
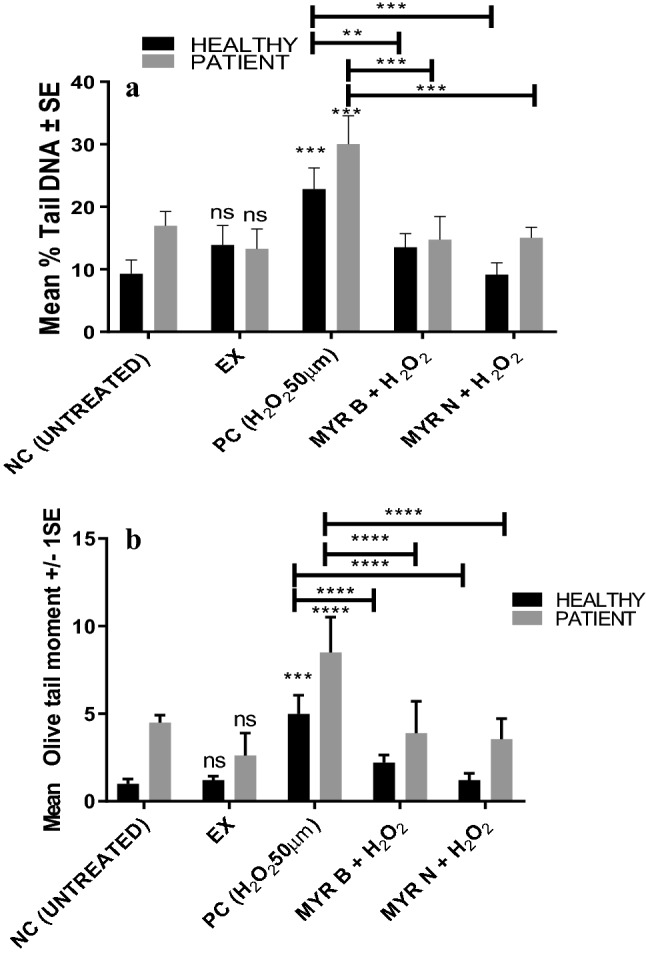
**a** Mean % tail DNA showing effect of MYR N & B on H_2_O_2_-induced DNA damage in lymphocyte taken from healthy individuals and pre-cancerous patients. Figure shows four groups of treatments including the negative control, excipient mixture, positive control, MYR B (10 µM) co-supplemented with H_2_O_2_ and MYR N (20 µM) co-supplemented with H_2_O_2_. All treatments were compared against the PC. The mean control values for the % tail DNA of NC and PC group for healthy individuals and patients were 9, 22 and 18, 29, respectively, measuring 100 cells each per experiment. Asterisk shows significant difference between the groups. The horizontal lines on top of the graph show the significance difference the positive control and the treatment groups. (For healthy groups **P < 0.009, ***P < 0.005. For patient groups ****P < 0.001, *** P< 0.003). **b** Mean OTM showing the effect of MYR B & N on H_2_O_2_-induced DNA damage in lymphocytes from healthy volunteers and pre-cancerous patients. Figure shows four groups of treatments including the negative control, positive control, MYR B (10 µM) co-supplemented with H_2_O_2_ and MYR N (20 µM) co-supplemented with H_2_O_2._ All treatment groups were compared to the PC group. The mean NC values for the OTM of healthy and patient groups were 0.9 and 4.5, respectively. The mean maximum values of the PC of healthy and patient groups were 5 and 8, respectively. The horizontal lines on top of the graph show the significance difference between the positive control and the treatment groups. (***P < 0.001, ***P < 0.007 shown on healthy groups and ***P < 0.002, ***P < 0.001 for patients groups)

In-vitro treatment of lymphocytes from healthy individuals and patients with MYR B (10 µM) and MYR N (20 µM), co-supplemented with 50 µM H_2_O_2_, resulted in significant reduction of DNA damage when compared to the PC (50 µM H_2_O_2_) alone. MYR N and NC show the same level of DNA damage in healthy lymphocytes both in %tail DNA and Olive tail moment (OTM). The H_2_O_2_-induced DNA damage was significantly reduced by MYR B and MYR N in both groups, whereas MYR N was more effective in healthy group when compared to the PC (Fig. 3a, b), giving the value almost similar to the untreated group.

### Antioxidant effects of myricetin

The study used a cell permeant reagent 2′,7′-dichlorofluorescin diacetate (DCFDA) that measures total intracellular ROS activity and levels. Once diffused into the cells, cellular esterases deacetylate DCFDA as a non-fluorescent form which is later oxidized to a highly fluorescent compound, 2′,7′-dichlorofluorescein (DCF), by interacting with ROS and easily detected by fluorescence spectroscopy. The effect of MYR B and MYR N on basal ROS was determined in lymphocytes from the healthy group and pre-cancerous patients. Results showed that MYR N has significantly reduced the ROS levels in lymphocytes from both groups.

### TBHP-induced oxidative injury in lymphocytes: protection by myricetin

**Fig. 4:**
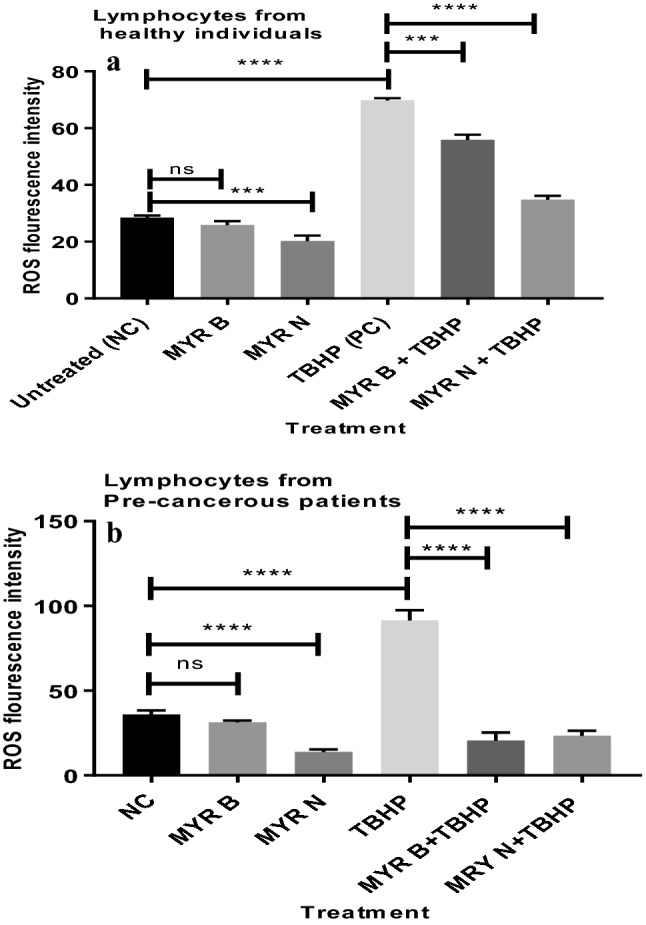
**a** Average of 3 independent experiments showing, change in intracellular ROS before and after treatment with TBHP in healthy lymphocyte. The horizontal lines show the significant difference between the groups. ***P < 0.001, *ns* not significant. Six treatment groups included an untreated group (NC), MYR B (10 µM), MYR N (20 µM), TBHP ((300 µM) as PC, TBHP + MYR B, and TBHP + MYR N. **b** Average of 3 independent experiments showing, change in intracellular ROS before and after treatment with TBHP in lymphocyte from pre-cancerous patients. The horizontal lines show the significant difference between the groups. *Ns* not significant, ***P < 0.001. Six treatment groups included an untreated group (NC), MYR B (10 µM), MYR N (20 µM), TBHP ((300 µM) as PC, TBHP + MYR B, and TBHP + MYR N

Exposure of healthy and patient lymphocytes to TBHP alone induced a significant increase in ROS levels by 40%. However, upon treatment with MYR B and MYR N, a significant attenuation of TBHP-induced ROS was observed in lymphocytes from both groups (Fig. 4a, b).

### Activity of intracellular antioxidant enzyme, GSH, and change in GSH/GSSG ratio

**Fig. 5:**
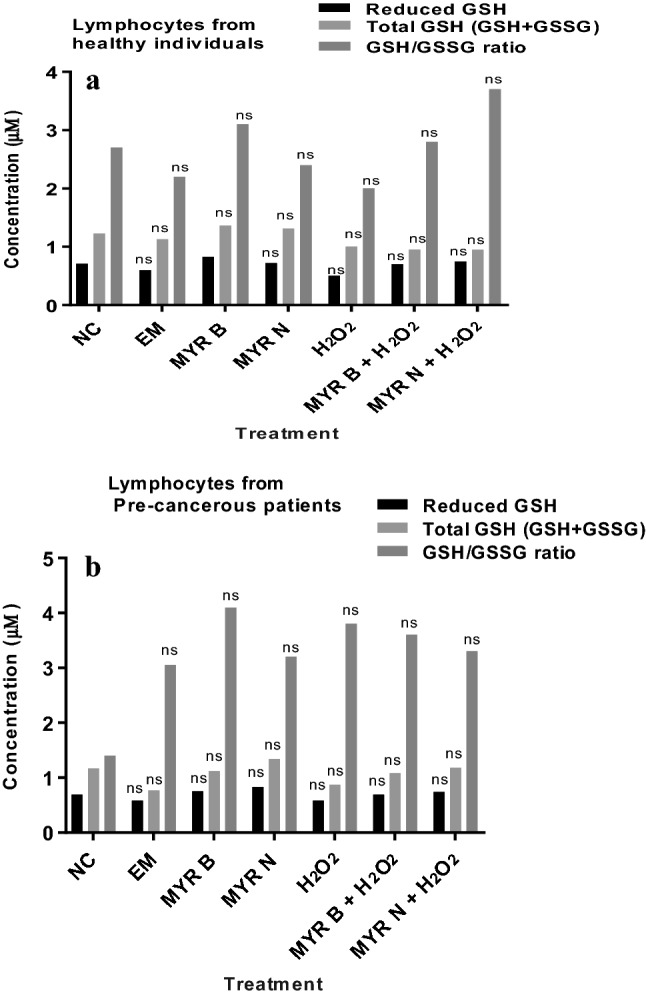
Levels of different forms of GSH in healthy lymphocyte (**a**) and those from pre-cancerous patients (**b**). Cells lysed to the concentration of 1 × 10^5^ cells/ml. Various treatment groups included the NC (untreated), EX, MYR B (10 µM), MYR N (20 µM), H_2_O_2_ (50 µM) as PC, MYR B + H_2_O_2_, and MYR N + H_2_O_2._*ns* not significant

The effects of MYR B and MYR N on the basal levels of GSH were assessed by treating lymphocytes from healthy and patient group with both forms of myricetin alone. MYR B and MYR N have shown a trend to increase the basal levels of GSH/GSSG ratio in lymphocytes from pre-cancerous patients, but levels are not significantly different. Hence, myricetin does not show any substantial effect on GSH levels in lymphocytes from healthy individuals and in those from pre-cancerous patients (Fig. 5a, b).

### Effect of myricetin on DSB formation in lymphocytes at basal levels

**Fig. 6:**
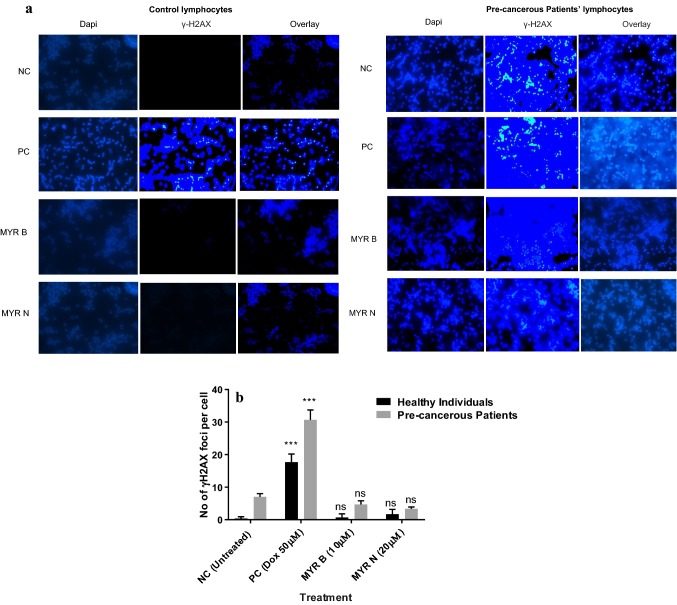
**a***γ*H2AX phosphorylation in lymphocytes from healthy individuals and pre-cancerous patients presented in untreated cells (NC) and treated with doxorubicin (50 μM), MYR B (10 Μm), and MYR N (20 μM). **a** Merged Dapi and gamma-H2AX stains in blood lymphocytes from healthy individuals and pre-cancerous patients. Number of phosphorylated H2AX-foci corresponds with DSBs. **b***γ*H2AX foci induction in lymphocytes with different treatment groups. Data were analysed by two-way ANOVA followed by multiple comparison test for significant differences compared to the untreated control for each group (***p < 0.001, *ns* not significant)

DSBs in the nuclei of lymphocyte cells were immunocytochemically stained using the *γ*H2AX protein, and *γ*H2AX foci inside each nucleus were counted in 100 cells each. One DSB represented one *γ*H2AX focus. Results showed that myricetin does not cause DSBs in healthy lymphocytes (Fig. 6a, b). Compared to the number of foci generated in the control, both forms of myricetin (MYR B and MYR N) have not shown any significant increase in foci formation. However, as shown in Fig. 6b, an increased number of *γ*H2AX foci formation was observed after treatment of the healthy lymphocytes with doxorubicin, a known strand break inducer (PC) (*p* < 0.001). Foci formation was observed at basal levels in untreated group from pre-cancerous patients and this incidence of the foci formation was significantly increased (*p* < 0.001) after doxorubicin treatments. Upon treatment with MYR B (10 µM) and MYR N (20 µM) forms in lymphocytes from pre-cancerous patients, no significant effect was observed in *γ*H2AX foci formation compared to the untreated group. This suggests that myricetin does not induce DSBs in healthy lymphocytes at basal levels and could potentially provide protection against DSBs formation in lymphocytes from pre-cancerous patients at basal levels.

## Discussion

In this study, we determined the effects of myricetin against H_2_O_2_-induced DNA damage by simultaneously exposing the cells to H_2_O_2_ (50 µM) in the presence or absence of MYR B or MYR N. The Comet assay results have shown the protective effect of myricetin bulk and nanoparticles (NPs) against H_2_O_2_-induced ROS-related oxidative damage in the lymphocytes of healthy individuals and pre-cancerous MGUS patients. The basal DNA damage was significantly inhibited when compared to the positive control (Fig. 3a, b). Both forms of myricetin have shown inhibitory effects, whereas MYR N was more effective in causing the reduction in DNA damage, may be due to its enhanced physio-chemical characteristics (*p* < 0.001). The potential explanation for this could be that NPs due to their minute size can easily reach the nucleus through diffusion across the nuclear membrane or transportation via the nuclear pores and gain direct interaction with the DNA (Magdolenova et al. 2012, 2014). This could be further investigated and confirmed by studying the sub-cellular distribution of these particles using transmission electron microscopy (TEM).

ROS play a crucial role in stimulating DNA strand breaks formation and causing oxidative stress (Tanaka et al. 2007). Accumulation of the DNA damage induced by ROS can lead to various deleterious processes including the stimulation of neoplasms production and eventually tumour development (Tanaka et al. 2007; Vilenchik and Knudson 2003). To evaluate the antioxidant potential of myricetin, we investigated its antioxidant effects in lymphocytes from healthy individuals and pre-cancerous MGUS patients by measuring intracellular ROS contents. The basal levels of ROS and the effect of myricetin against TBHP-induced ROS were evaluated. Results have demonstrated that myricetin has reduced the basal damage in lymphocytes from both the investigated groups as well as the insults introduced by TBHP treatment (Fig. 4a, b). Both forms of myricetin have shown strong antioxidant defence by scavenging the free radicals caused by ROS and by reducing their levels intracellularly. However, when two forms were compared, MYR N (20 µM) demonstrated more protective effects than MYR B (10 µM). Our results are consistent with previous studies (Wang et al. 2010; Kang et al. 2010). Inhibition of ROS may prevent its accumulation, also the interaction of floating radicals with cellular contents and DNA fragmentation, avoiding DNA lesions and strand breaks. Hence, myricetin shows anti-mutagenic and anti-carcinogenic properties by preventing mutations at non-genotoxic concentrations used for both forms of myricetin. These results are consistent with previous studies conducted on various cells lines using myricetin. It has been previously shown that myricetin protects cells from H_2_O_2_ damage by inhibiting ROS production and by stimulating the antioxidant enzymes. It restored the function of antioxidant defence enzymes such as catalase (CAT), superoxide dismutase (SOD), and glutathione peroxidase (GPx) which was reduced by H_2_O_2_ treatment (Wang et al. 2010).

Although we used the fluorescent probe DCFDA to detect ROS levels, the formamido pyrimidine glycosylase (FPG) protein could also be considered to assess oxidative DNA damage using the Comet assay (Müller et al. 2013).

Furthermore, this study also investigated the effect of myricetin on intracellular glutathione levels. In healthy cells, the total glutathione pool is mostly in the reduced form (GSH), a key tissue antioxidant that presents first line of defense against ROS. However, when cells are exposed to increased levels of oxidative stress, oxidized glutathione (GSSG) starts gathering and the ratio of GSSG to GSH rises. Hence, a bigger ratio of GSSG-to-GSH is an indication of oxidative stress (Roy and Sil 2012). Our results have demonstrated no significant effect on GSH levels after treatment with MYR B and MYR N in lymphocytes from healthy and MGUS patient groups (Fig. 5a, b).

Gamma-H2AX is used as an effective biomarker of DSBs. An accumulating body of evidence suggests that the crucial role of *γ*H2AX phosphorylation for nuclear foci formation at DBS sites and stimulation of DNA repair (Rogakou et al. 1998; Podhorecka et al. 2010). There exists a one-to-one correspondence between DSBs and gamma-H2AX foci; hence, DSBs can be easily visualised immunocytochemically (Rogakou et al. 1999). The number of foci, therefore, could be used as a relative parameter to estimate DNA damage and repair. To evaluate the effect of myricetin on DSBs formation in lymphocytes from healthy individuals and those from pre-cancerous MGUS patients, we quantified *γ*H2AX foci intensity using immunofluorescence. Our results have shown that myricetin does not induce DSBs formation in healthy lymphocyte and those from the pre-cancerous patients at basal levels (Fig. 6a, b) which is consistent with our previous results from the Comet assay where no significant DNA damage was caused upon exposure to MYR B (10 µM) and MYR N (20 µM). However, a trend to lower an overall intensity of *γ*H2AX foci was observed in the lymphocytes from pre-cancerous MGUS patients treated with MYR B and MYR N when compared to the untreated group. This marks the current study as the first one to demonstrate the effect of MYR B (10 µM) and MYR N (20 µM) on DSBs development in lymphocytes from healthy individuals and those from pre-cancerous MGUS patients at basal levels.

In conclusion, this work demonstrates for the first time (to our knowledge) that myricetin bulk and nano at selective non-genotoxic concentrations protect the lymphocytes (from healthy individuals and pre-cancerous patients) from damaging effects of TBHP and H_2_O_2_ primarily by inhibiting ROS-induced oxidative stress. Besides this, MYR B (10 µM) and MYR N (20 µM) do not induce formation of DSBs in lymphocytes from healthy individuals and pre-cancerous MGUS patients at basal levels and could possibly protect the lymphocytes from extensive cell damage by inhibiting DSBs formation and ultimately help in cell survival and DNA damage repair. DNA repair capacity of lymphocytes after treatment with myricetin could possibly be studied using the Comet assay simply by assessing the damage at different time periods (Collins 2004). The overall results have demonstrated that MYR N (20 µM) has shown better antioxidant and genoprotective effects against the oxidative damage in lymphocytes from healthy individuals and pre-cancerous MGUS patients when compared to MYR B (10 µM).
